# Nutritional Value of the Duckweed Species of the Genus *Wolffia* (Lemnaceae) as Human Food

**DOI:** 10.3389/fchem.2018.00483

**Published:** 2018-10-29

**Authors:** Klaus-J. Appenroth, K. Sowjanya Sree, Manuela Bog, Josef Ecker, Claudine Seeliger, Volker Böhm, Stefan Lorkowski, Katrin Sommer, Walter Vetter, Karla Tolzin-Banasch, Rita Kirmse, Matthias Leiterer, Christine Dawczynski, Gerhard Liebisch, Gerhard Jahreis

**Affiliations:** ^1^Matthias Schleiden Institute, Plant Physiology, Friedrich Schiller University Jena, Jena, Germany; ^2^Department of Environmental Science, Central University of Kerala, Kasaragod, India; ^3^Institute of Botany and Landscape Ecology, University of Greifswald, Greifswald, Germany; ^4^Chair of Nutrition Physiology, University Munich, Freising, Germany; ^5^Institute of Nutritional Sciences, Friedrich Schiller University Jena, Jena, Germany; ^6^Competence Cluster for Nutrition and Cardiovascular Health (nutriCARD) Halle-Jena-Leipzig, Jena, Germany; ^7^Institute of Food Chemistry, University of Hohenheim, Stuttgart, Germany; ^8^Thuringian State Institute of Agriculture, Jena, Germany; ^9^Institute of Clinical Chemistry and Laboratory Medicine, University Hospital Regensburg, Regensburg, Germany

**Keywords:** amino acids, duckweed, fatty acids, Lemnaceae, phytosterols, protein, *Wolffia*

## Abstract

Species of the genus *Wolffia* are traditionally used as human food in some of the Asian countries. Therefore, all 11 species of this genus, identified by molecular barcoding, were investigated for ingredients relevant to human nutrition. The total protein content varied between 20 and 30% of the freeze-dry weight, the starch content between 10 and 20%, the fat content between 1 and 5%, and the fiber content was ~25%. The essential amino acid content was higher or close to the requirements of preschool-aged children according to standards of the World Health Organization. The fat content was low, but the fraction of polyunsaturated fatty acids was above 60% of total fat and the content of n-3 polyunsaturated fatty acids was higher than that of n-6 polyunsaturated fatty acids in most species. The content of macro- and microelements (minerals) not only depended on the cultivation conditions but also on the genetic background of the species. This holds true also for the content of tocopherols, several carotenoids and phytosterols in different species and even intraspecific, clonal differences were detected in *Wolffia globosa* and *Wolffia arrhiza*. Thus, the selection of suitable clones for further applications is important. Due to the very fast growth and the highest yield in most of the nutrients, *Wolffia microscopica* has a high potential for practical applications in human nutrition.

## Introduction

Duckweeds represent a small family of aquatic floating monocots consisting of 37 species distributed all over the world (Landolt, 1986; Sree et al., 2016). These plants are the fastest growing angiosperms (Sree et al., 2015; Ziegler et al., 2015) and may cover ponds or lakes within a few days under favorable growth conditions. It is frequently observed that animals, such as ducks, swans, or geese, feed on duckweeds growing naturally in ponds or lakes. Of course, this is where the name, duckweed, comes from. These plants have also been used for a long time to feed domesticated animals, either by providing them temporary access to duckweed grown ponds or by supplementing their diet with harvested duckweed, fresh or dried; in case of pigs, it has been already reported in the 1960s, and later reports with cattle, rams, sheep, horses, waterfowls, and fishes have been detailed by Landolt (1986). The World Bank had also supported a project to feed fish with duckweeds in Bangladesh (Skillicorn et al., 1993). More recently, detailed reports were published in this regard, e.g., using *Wolffia arrhiza* meal as a substitute for soya in the diet of Japanese quails (Suppadit et al., 2012), using duckweed species in the feed of striped catfish (Da et al., 2013), of rohu and carp (Sharma et al., 2016), of broilers (Shammout and Zakaria, 2015), and use of genetically modified *Lemna minor* to feed laying hens (Ghosh et al., 2015).

Duckweeds, in several Asian countries, also serve as human food. With the local names of *khai nam, kai-pum*, or *kai nhae* (literally meaning: water-eggs) the rootless duckweed *Wolffia globosa* is sold in the vegetable markets in different regions of Thailand. Bhanthumnavin and McGarry (1971) and Rusoff et al. (1980) investigated some of the duckweed species and suggested them as a possible source of protein. In these countries, fresh *Wolffia* plants are used to prepare several dishes like salads, omelets or vegetable curries (Saengthongpinit, 2017). Protein quantity and quality are important features especially in those countries with starch-rich staple food like rice and maize (Appenroth et al., 2017). In a recent publication, we provided an overview of the nutritional status of the whole plant family, Lemnaceae, and investigated species from each of the five genera concerning their protein content, amino acid spectrum, starch content, fat content, and fatty acid distribution (Appenroth et al., 2017). Moreover, we selected the fastest growing species, *Wolffia microscopica* to investigate the mineral composition, phytosterols and fiber content. From the set of species selected for the study, *W. microscopica* and *Wolffiella hyalina* were the most promising candidates with respect to their nutritional value (Appenroth et al., 2017). *W. microscopica* is an endemic species to the Indian subcontinent and *W. hyalina* has a closed distribution pattern. However, some of the other species of the genus *Wolffia* have a widespread distribution. *W. globosa* is a species mostly used for human nutrition in the Asian countries. Unlike the species belonging to the subfamily Lemnoideae (i.e., *Spirodela, Landoltia*, and *Lemna*), species of the genus *Wolffia* have the advantage that the oxalate content is not present in the form of calcium oxalate crystals that might cause health problems to humans (Landolt and Kandeler, 1987). In our previous paper, where it has been demonstrated that some of the duckweed species have excellent qualities for human nutrition, these species were selected as representatives of the duckweed genera. In the present paper, we addressed the question whether different species of the same genus contain similar or varying nutritional qualities concerning human nutrition by investigating all the 11 existing species of the genus *Wolffia* with a prime focus on *W. globosa*.

## Materials and methods

### Plant material and cultivation

Plant material was taken from the collection of duckweed strains, or clones, of the Department of Plant Physiology, University of Jena, Germany. The duckweeds in this collection, most of which stem from the collection of Prof. Elias Landolt, ETH, Zurich, Switzerland, were maintained under axenic conditions as described before (Appenroth et al., 1996). All 11 species of the genus *Wolffia* were represented by one clone each. The species *W. globosa* and *W. arrhiza*, however, were additionally represented by four and three clones, respectively (Table 1).

**Table 1:** List of all 11 species of the genus *Wolffia* used in the present investigations.

**Genus**	**Species**	**Clone**	**Origin**	**Doubling time (d)**
*Wolffia*	*angusta*	8878	Malaysia	1.3 ± 0.2
*Wolffia*	*arrhiza*	8618	Kenya	1.6 ± 0.1
		8853	Brazil	2.3 ± 0.0
		9528	Germany	1.8 ± 0.0
*Wolffia*	*australiana*	7540	New Zealand	1.4 ± 0.0
*Wolffia*	*borealis*	9123	USA	1.6 ± 0.0
*Wolffia*	*brasiliensis*	7925	Argentina	1.4 ± 0.1
*Wolffia*	*columbiana*	7155	USA	2.3 ± 0.1
*Wolffia*	*cylindracea*	9056	Zimbabwe	2.2 ± 0.1
*Wolffia*	*elongata*	9188	Colombia	1.6 ± 0.0
*Wolffia*	*globosa*	5514	Thailand	2.0 ± 0.0
		5515	Thailand	2.2 ± 0.1
		5537	Thailand	1.8 ± 0.2
		9498	India	1.2 ± 0.0
*Wolffia*	*microscopica*	2005	India	1.0 ± 0.0
*Wolffia*	*neglecta*	9149	Pakistan	1.2 ± 0.1

*Beside clone identity number, origin of the clones and growth rates, given as doubling time ± SD (for definition cf. Ziegler et al., 2015), are presented. d, days*.

Determination of *Wolffia* species on morphological basis alone is very difficult and sometimes not reliable (Landolt, 1994). Hence, the identity of each clone was confirmed by barcoding using several plastidic sequences (Bog et al., 2013; Supplementary Table S1) as suggested by Borisjuk et al. (2015). The plants were pre-cultivated and cultivated as described before (Appenroth et al., 2017), except that the modified Schenk-Hildebrandt medium was replaced by a modified Steinberg medium with increased Fe^3+^ and EDTA concentrations as this medium turned out to guarantee more stable cultivation of *Wolffia* plants with fewer infections in the 15 L-plastic trays. The composition of this medium was as follows (Appenroth, 2015): KNO_3_ 3.46 mM, KH_2_PO_4_ 0.66 mM, K_2_HPO_4_ 72 μM, MgSO_4_ 0.41 mM, Ca(NO_3_)_2_ 1.25 mM, H_3_BO_3_ 1.94 μM, ZnSO_4_ 0.63 μM, Na_2_MoO_4_ 0.18 μM, MnCl_2_ 0.91 μM, Fe(III)NaEDTA 14.05 μM, EDTA-Na_2_ 6.1 μM, pH was adjusted to 5.5. Because of the higher requirement of fresh weight for all analyses, the cultivation time was extended to 2–3 weeks.

Growth rates of all plants were determined and weekly yield (using the parameter fresh weight, FW) was calculated based on the exponential law defined as yield after seven days of cultivation starting with 1 g FW as described by Ziegler et al. (2015).

### Analytical methods

After freeze-drying of fresh duckweeds, the freeze-dry weight (FDW) was determined. The freeze-dried material was finely ground and homogenized with a laboratory mill and aliquoted for further analyses. All analyses were carried out using the same freeze-dried material and the data were related to FDW.

The analyses of dry matter, ash, total fat, and total protein were carried out as described before (Appenroth et al., 2017). Further components were analyzed as described below.

Starch content: The starch content was measured following acidic extraction and Lugol's staining (Appenroth et al., 2010).

Total fiber content: The content of total fiber was enzymatically determined according to the supplier's protocol (α-amylase, protease, amyloglucosidase; BIO-QUANT, Total Dietary Fiber, Merck).

Amino acids: Amino acids (AA) were determined after subjecting the samples to acid hydrolysis with phenolic hydrochloric acid. For the sulfur-containing amino acids, methionine and cysteine, an oxidation step was performed before acid hydrolysis. The analysis of AA was carried out by means of ion exchange chromatography (Biochrom 30 Plus, Laborservice Onken, Gründau, Germany) and post column derivatization with ninhydrin.

Fatty acids (FA): Analyses were performed as described previously (Ecker et al., 2012). Briefly, fatty acid methyl esters (FAMEs) were generated by acetyl chloride and methanol treatment and extracted with *n*-hexane. Total FA analysis was generally carried out using a Shimadzu 2010 GC/MS system. FAMEs were separated on a BPX70 column (10-m length, 0.10-mm internal diameter, 0.20-μm film thickness) from SGE using helium as carrier gas. The initial oven temperature was 50°C and was programmed to increase at 40°C/min to 155°C, 6°C/min to 210°C, and finally 15°C/min to 250°C. Two different methods were used to analyze FAMEs to achieve maximum analytical coverage. The FA species and their positional and *cis/trans* isomers were characterized in scan mode and quantified by single ion monitoring to detect specific fragments of saturated and unsaturated FAs (saturated, *m/z* 74; monounsaturated, *m/z* 55; diunsaturated, *m/z* 67; polyunsaturated, *m/z* 79). The internal standard was non-naturally-occurring C21:0 *iso* without stearidonic acid. For the determination of steridonic acid an additional run was carried out. FAMEs were separated using gas chromatography (GC-17A with flame ionization detector, Shimadzu, Japan) and a medium polar column (DB-225MS, Agilent Technologies, USA). The standard FAME mixture for this run contained identical stearidonic acid (SDA; C18:4-c6,9,12,15). To calculate the fatty acid percentage, the concentration of each detected FAME species was divided through the total concentration of FAMEs before multiplication with 100, i.e., ([c]FAME species/ [c]total FAME)^*^100.

Minerals: After microwave pressure acid digestion, macro elements and most of the trace elements were determined by inductively coupled plasma atomic emission spectroscopy (iCAP 6000, Thermo Fisher Scientific). For digestion 0.2 g of the ground, freeze-dried material were weighed in a quartz vessel and 2 ml of ultrapure water and 5 ml of 65% HNO_3_ were added. The vessels were closed and heated in the microwave digestion system (turboWAVE, Milestone S.r.L.) for 25 min (600–1,200 W). The digestion solution was filled up to 15 ml with ultrapure water. For the quantitative measurement of arsenic and selenium, the method of hydride generation atomic absorption spectrometry (PinAAcle 500, PerkinElmer) was used. The mercury content was determined from the solid matter by direct mercury analyzer (DMA-80, Milestone S.r.L.). For the analysis of iodine, a strong alkaline digestion of the freeze-dried material with aqueous solution of tetramethylammonium hydroxide (TMAH) came to use (0.5 g/5 ml ultrapure H_2_O/ml 25% TMAH). Measurement was carried out by inductively coupled plasma mass spectroscopy (NexION 350X, PerkinElmer). All quantifications were performed as independent duplicate analysis. The relative standard deviation (RSD) for all parameters was determined from independent repeat measurements of the control sample over a period of several months.

Antioxidants: Carotenoids and tocopherols were extracted and determined as recently described (Appenroth et al., 2017).

Phytosterols: Sterols were extracted and determined, in duplicate, using GC/MS according to Appenroth et al. (2017) with the following modifications. The silylated unsaponifiable matter was analyzed with a Thermo Scientific Trace GC/Trace DSQ MS system equipped with a split/splitless injector and a CTC PAL auto sampler. Sample injection (1 μL) was performed in splitless mode at 250°C. The initial carrier gas flow was set to 1 mL/min. After 0.1 min, a steep ramp of 500 mL/min/min was used to obtain a flow of 10 mL/min which was held for 1 min. Subsequently, the flow was reduced at 5 mL/min/min to 4 mL/min (hold time 2 min) and then at 1 mL/min/min to 1 mL/min which was maintained throughout the run. An HP5-MS UI column (30-m length × 0.25-mm internal diameter, 0.25-μm film thickness, Agilent Technologies) was used in combination with the following GC oven program. After 1 min at 55°C, temperature was raised at 25°C/min to 255°C, then at 1.5°C/min to 283°C and finally at 15°C/min to 300°C which was held for 8 min. Temperature of the transfer line was set to 280°C. Data were recorded from *m/z* 50 to 650 after a solvent delay of 7 min. Sterol separation was obtained by temperature program, whereas carrier gas flow program was used for the simultaneous determination of phytol and dihydrophytol. Total sterol content was calculated by internal standard calibration via 5α-cholestane. For the quantification of the phytols an external calibration curve was used.

### Statistics

Lyophilized plant material from a single batch was used for all investigations. All data based on two independent measurements (*n* = 2). As reference systems, either the freeze-dry weight (content of protein, fat, fiber, minerals, carotenoids, tocopherol), or the total protein content (amino acids), or the total fat content (fatty acids, sterols) were used. The RSD gives the matrix-specific precision (repeatability) of the analytical procedure in percent under run-to-run conditions determined by long-term experiments (Ecker et al., 2012). Averages ± standard deviations (SD) are given for each amino acid, fatty acid, minerals, tocopherol, carotenoids, and phytosterols of all 16 *Wolffia* clones.

## Results

### Overview of nutritional values of species and clones

**Figure 1:**
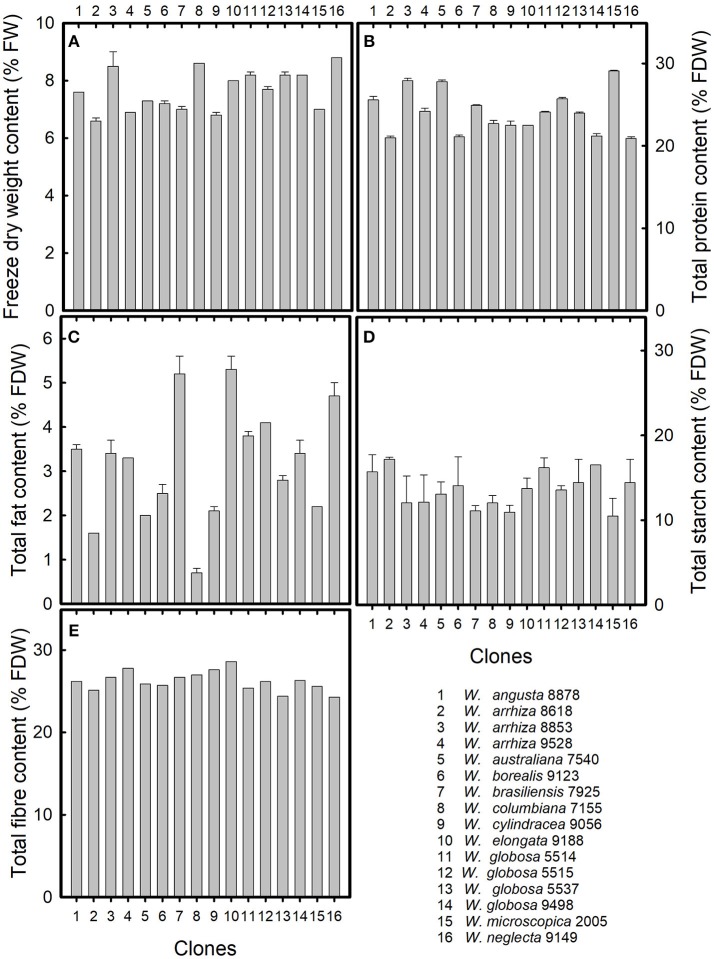
Chemical composition of 11 species of the genus *Wolffia*. **(A)** Freeze dry weight in relation to fresh weight, and **(B)** total protein content, **(C)** total fat content, **(D)** total starch content, and **(E)** total fiber content in relation to freeze dry weight. Data were given as means together with standard deviations of parallel measurements. The numbers on the x-axis represent the species investigated. For further explanations, see Table 1.

In the freeze-dried material (about 92% dry weight, e.g., about 8% remaining water) of the eleven species of the genus *Wolffia*, the contents of protein, fat, starch, and fiber were determined (Figure 1). Because of the frequency of distribution and their traditional practical use in human nutrition, *W. globosa* and *W. arrhiza* were represented by four and three clones, respectively. The freeze dry weight of the freshly harvested plant material was between ~7 and 9% of the fresh weight (Figure 1A), the starch content was between 10 and 15% of FDW (Figure 1D), and the fiber content was about 25% of FDW (Figure 1E). Larger variations between the plant samples were detected for the protein content, varying between 20 and 30% (Figure 1B). The largest differences were found for total fat content (Figure 1C), varying between 0.7% in *W. columbiana* and 5.3% in *W. elongata*.

### Amino acid distribution

**Figure 2:**
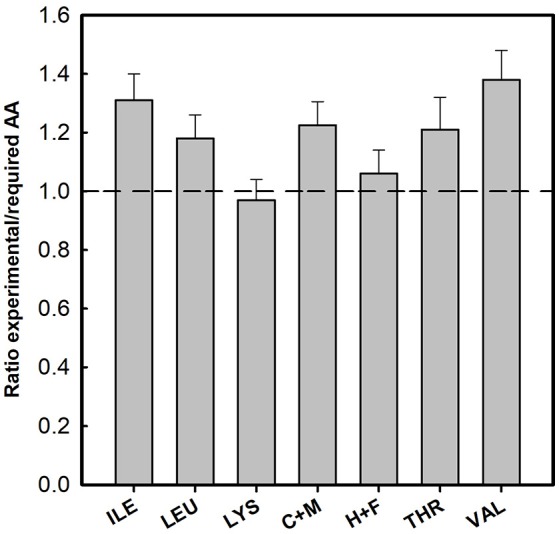
Ratio between the essential amino acid content in duckweed clones and requirements in preschool-age children (WHO (World Health Organization), 1985). C+M, cysteine + methionine; H+F, histidine + phenylalanine. Data were given as average of all investigated clones (± SD), the data for the single *Wolffia* clones were given in the Supplementary Material (Table S2).

The AA content of all investigated samples for 17 AA is given in Table 2. Especially for human nutrition, cysteine + methionine, threonine, phenylalanine, tyrosine, lysine, and leucine are important. Therefore, we compared the contents of critical AA with the reference pattern based on the essential amino acid requirements of the preschool-age child as published in 1985 [WHO (World Health Organization), 1985]. Figure 2 reports the ratio between the measured AA content of the investigated clones (averages) with those of the reference (for details see Supplementary Table S2). In all samples, this ratio for isoleucine, leucine, cysteine + methionine, threonine, and valine was above 1. Lysine and the aromatic AA histidine + phenylalanine (H+F) were slightly limiting AA in duckweeds. Most of the ratios for lysine were close to 1 (*W. angusta* 8878, *W. arrhiza* 8618, *W. borealis* 9123, *W. brasiliensis* 7925, *W. cylindracea* 9056*, W. microscopica* 2005, and *W. neglecta* 9149; see Supplementary Table S2). In some cases, the ratio was slightly lower than 1, with the lowest value of 0.84 for *W. arrhiza* 9528. For the aromatic AA, histidine and phenylalanine, four of the duckweed clones had ratios slightly below 1 (between 0.92 and 0.97). The average of AA showed that practically all duckweed clones fulfilled the requirement for human nutrition (Figure 2). Several species had ratios above 1 compared to the reference protein.

**Table 2:** Amino acid composition of protein of *Wolffia* species (g/100 g protein) (description of clones, Table 1).

**Clone**	**Ala**	**Arg**	**Asp**	**Cys**	**Glu**	**Gly**	**His**	**Ile**	**Leu**	**Lys**	**Met**	**Phe**	**Pro**	**Ser**	**Thr**	**Tyr**	**Val**
*W. angusta* 8878	7.1	6.0	13.3	1.7	12.1	5.2	1.7	3.9	8.4	6.0	1.7	5.0	4.5	5.3	4.4	3.2	5.0
*W. arrhiza* 8618	6.7	5.9	9.6	1.3	11.7	5.2	1.9	3.8	8.3	6.0	1.6	5.2	4.6	4.8	4.0	3.3	5.0
*W. arrhiza* 8853	8.8	5.8	16.3	1.5	11.0	5.2	1.7	3.3	7.2	5.4	1.4	4.4	4.2	5.4	3.8	3.1	4.4
*W. arrhiza* 9528	8.8	5.4	13.0	1.4	10.8	5.2	1.7	3.3	7.1	4.9	1.4	4.3	4.0	5.4	3.6	2.7	4.5
*W. australiana* 7540	6.3	5.5	15.5	1.4	11.1	5.0	1.8	3.6	7.7	5.6	1.5	4.8	4.0	4.6	4.1	3.3	4.7
*W. borealis* 9123	6.6	5.8	10.4	1.4	11.4	5.3	1.9	3.8	8.0	6.0	1.7	5.0	4.7	4.6	4.2	3.5	5.1
*W. brasiliensis* 7925	6.6	6.4	13.5	1.6	12.9	5.0	1.9	3.7	8.1	6.0	1.5	4.9	4.3	4.7	4.0	3.5	5.1
*W. columbiana* 7155	6.7	5.3	11.4	1.7	11.8	5.7	1.6	3.2	7.2	5.2	1.3	4.5	3.8	5.5	3.4	2.8	4.3
*W. cylindracea* 9056	6.4	5.7	9.3	1.6	11.0	5.3	1.7	3.6	7.7	5.9	1.7	4.7	4.3	5.0	3.9	2.8	4.8
*W. elongata* 9188	6.6	6.2	9.6	1.4	11.2	5.4	2.2	3.9	8.6	5.7	1.7	5.3	4.8	5.2	4.6	3.6	5.1
*W. globosa* 5514	6.4	5.8	12.1	1.6	11.0	5.1	1.8	3.7	7.7	5.4	1.7	4.9	4.6	4.5	4.5	3.5	4.8
*W. globosa* 5515	6.5	5.8	14.0	1.5	10.8	4.7	1.6	3.3	6.9	5.0	1.5	4.2	4.0	4.2	3.9	3.1	4.3
*W. globosa* 5537	6.9	5.6	9.7	1.5	11.1	5.2	1.7	3.6	7.5	5.4	1.6	4.6	4.4	4.5	4.2	3.3	4.6
*W. globosa* 9498	6.4	5.8	10.8	1.6	11.0	5.5	2.0	3.8	7.7	5.6	1.5	4.7	4.6	5.0	4.2	3.4	4.9
*W. microscopica* 2005	6.5	5.8	12.8	1.6	12.2	5.2	1.8	3.8	8.1	6.0	1.7	4.8	4.3	5.0	4.5	3.2	5.0
*W. neglecta* 9149	6.4	5.9	10.6	1.6	11.4	5.6	1.9	3.7	7.9	6.1	1.7	4.7	4.6	5.7	4.3	3.4	5.1
RSD (%)	1.7	1.2	1.5	3.4	2.9	1.6	2.4	2.9	2.2	2.5	3.7	3.8	4.3	2.9	3.1	3.0	1.6
Average	6.9	5.8	12.0	1.5	11.4	5.2	1.8	3.6	7.8	5.6	1.6	4.7	4.4	5.0	4.1	3.2	4.8
± SD	0.8	0.3	2.2	0.1	0.6	0.2	0.2	0.2	0.5	0.4	0.1	0.3	0.3	0.4	0.3	0.3	0.3

*Relative standard deviation (RSD) gives the matrix-specific accuracy (repeatability) of the analytical procedure in percent. Mean and standard deviation (SD) characterize the average content and variation of each analyzed amino acid across all 16 clones*.

### Fatty acid distribution

The fat in duckweed contained ~34% saturated fatty acids with particularly high levels of palmitic acid (C16:0). Capric acid (C10:0), myristic acid (C14:0), margaric acid (C17:0), stearic acid (C18:0) and long-chain (>C18:0) saturated fatty acids (SFA) were present in rather small amounts or only in traces (Table 3). The highest fraction of SFA (Tables 3, 5) was detected in *W. globosa* 9498 (~42%), the lowest was in *W. arrhiza* 9528 (~29%).

**Table 3:** Distribution of saturated fatty acids in lipids of *Wolffia* species [% of FAME].

**Clone**	**C10:0**	**C14:0**	**C16:0**	**C17:0**	**C18:0**	**C20:0**	**C22:0**	**C24:0**	**C26:0**	**C28:0**
*W. angusta* 8878	0.13	0.33	30.0	0.29	2.2	0.68	0.36	0.63	0.55	0.24
*W. arrhiza* 8618	0.26	0.40	27.7	0.22	2.0	0.69	0.71	0.38	0.37	0.26
*W. arrhiza* 8853	0.12	0.34	30.8	0.23	1.8	0.42	0.55	0.39	0.39	0.32
*W. arrhiza* 9528	0.21	0.47	24.6	0.20	1.7	0.65	0.66	0.38	0.32	0.17
*W. australiana* 7540	0.20	0.41	28.4	0.19	2.2	0.62	0.85	1.50	0.44	0.13
*W. borealis* 9123	0.14	0.31	25.6	0.18	2.0	0.45	0.27	0.40	0.38	0.15
*W. brasiliensis* 7925	0.18	0.31	32.3	0.24	1.9	0.85	0.59	0.84	0.24	0.06
*W. columbiana* 7155	0.19	0.23	26.6	0.16	2.0	0.58	0.62	0.62	0.33	0.07
*W. cylindracea* 9056	0.18	0.44	29.9	0.23	1.9	0.60	0.67	0.43	0.35	0.22
*W. elongata* 9188	0.15	0.57	27.6	0.25	2.2	0.33	0.60	0.34	0.52	0.28
*W. globosa* 5514	0.20	0.30	30.0	0.22	2.3	0.40	0.24	0.43	0.40	0.17
*W. globosa* 5515	0.17	0.28	27.8	0.21	2.6	0.45	0.26	0.46	0.38	0.14
*W. globosa* 5537	0.17	0.41	28.1	0.20	2.1	0.43	0.26	0.49	0.40	0.14
*W. globosa* 9498	0.34	0.55	34.3	0.35	3.6	0.75	0.64	0.54	0.59	0.34
*W. microscopica* 2005	0.14	0.59	26.8	0.13	1.8	0.47	0.00	0.05	1.18	0.00
*W. neglecta* 9149	0.23	0.37	28.0	0.24	2.6	0.45	0.29	0.31	0.36	0.19
RSD (%)	10,2	4,4	6,2	5,5	5,8	9,4	7,5	6,5	9,5	9,5
Average	0.19	0.39	28.7	0.22	2.2	0.55	0.47	0.51	0.45	0.18
± SD	0.06	0.11	2.5	0.05	0.5	0.15	0.23	0.31	0.21	0.08

*For further explanations see Table 2*.

The monounsaturated fatty acids (MUFA), oleic acid (C18:1-c9), *cis*-vaccenic acid (C18:1-c11), and gondoic acid (C20:1-c11), were present (~2 – 4%) in smaller amounts than SFA (Tables 4, 5). *Wolffia elongata* 9188 comprised the highest MUFA fraction with 7.4%.

**Table 4:** Distribution of mono- and polyunsaturated fatty acids in lipids of *Wolffia species* [% of FAME].

**Clone**	**C18:1-c9**	**C18:1-c11**	**C18:2-c9,12**	**γC18:3-c6,9,12**	**αC18:3-c9,12,15**	**C20:1-c11**	**C20:2-c11,14**
	**n-9**	**n-7**	**n-6**	**n-6**	**n-3**	**n-9**	**n-6**
*W. angusta* 8878	3.47	0.23	20.5	n.d.	40.1	0.16	0.08
*W. arrhiza* 8618	2.54	0.42	25.9	n.d.	37.8	0.06	0.10
*W. arrhiza* 8853	2.58	0.51	28.7	n.d.	32.5	0.16	0.13
*W. arrhiza* 9528	2.00	0.59	24.6	n.d.	42.9	0.17	0.16
*W. australiana* 7540	1.91	0.80	19.1	1.01	42.0	0.08	0.10
*W. borealis* 9123	2.73	0.83	26.0	0.07	40.2	0.15	0.13
*W. brasiliensis* 7925	1.52	0.53	23.3	0.47	36.6	0.02	0.06
*W. columbiana* 7155	1.62	0.43	23.0	0.75	42.4	0.16	0.16
*W. cylindracea* 9056	2.76	0.53	24.9	n.d.	36.6	0.17	0.08
*W. elongata* 9188	6.18	1.03	27.5	1.38	30.7	0.16	0.09
*W. globosa* 5514	2.63	1.33	24.1	0.03	37.0	0.13	0.10
*W. globosa* 5515	2.95	0.78	25.9	0.03	37.4	0.16	0.10
*W. globosa* 5537	3.07	0.89	26.0	0.34	36.8	0.15	0.10
*W. globosa* 9498	2.55	0.80	26.1	0.22	28.1	0.13	0.09
*W. microscopica* 2005	1.20	0.50	26.2	1.60	37.0	0.09	0.09
*W. neglecta* 9149	2.76	0.75	27.6	0.21	35.4	0.14	0.11
RSD (%)	5,8	3,8	6,1	4,2	5,5	8,1	6,6
Average	2.65	0.68	25.0	0.38	37.1	0.13	0.10
± SD	1.12	0.27	2.5	0.53	4.1	0.04	0.03

*n.d., not detectable. The limit of detection (LOD) was calculated as three times the noise signal of a blank. For further explanations see Table 2*.

**Table 5:** Fatty acid groups and n-6/n-3 ratio in lipids of *Wolffia* species [% of FAME].

**Clone**	**Sum SFA**	**Sum MUFA**	**Sum PUFA**	**Sum n-3**	**Sum n-6**	**n-6/n-3**
*W. angusta* 8878	35.4 (0.6)	3.86 (0.8)	60.8 (0.2)	40.2 (0.2)	20.6 (0.5)	0.51
*W. arrhiza* 8618	33.1 (0)	3.02 (0.7)	63.9 (0.3)	37.9 (0.3)	26.0 (0.4)	0.69
*W. arrhiza* 8853	35.4 (0.6)	3.26 (0.6)	61.4 (0.3)	32.6 (0.6)	28.8 (0.3)	0.89
*W. arrhiza* 9528	29.4 (0.7)	2.77 (8.7)	67.8 (0.3)	43.0 (0.2)	24.8 (0.4)	0.58
*W. australiana* 7540	34.9 (0.3)	2.79 (0.4)	62.3 (0.2)	42.1 (0.2)	20.2 (0)	0.48
*W. borealis* 9123	28.9 (1.4)	3.70 (0.3)	66.4 (0.6)	40.2 (0.5)	26.2 (0.8)	0.65
*W. brasiliensis* 7925	37.5 (1.3)	2.07 (7.0)	60.4 (1.0)	36.6 (1.1)	23.8 (0.8)	0.65
*W. columbiana* 7155	31.4 (1.3)	2.21 (1.4)	66.4 (0.5)	42.5 (0.7)	23.9 (0.4)	0.56
*W. cylindracea* 9056	34.9 (0)	3.46 (1.2)	61.6 (0)	36.7 (0)	25.0 (0)	0.68
*W. elongata* 9188	32.9 (0.6)	7.37 (1.2)	59.8 (0.2)	30.8 (0.3)	29.0 (0.3)	0.94
*W. globosa* 5514	34.7 (0.3)	4.09 (3.4)	61.2 (0.3)	37.0 (0.3)	24.2 (0.4)	0.66
*W. globosa* 5515	32.7 (0.9)	3.88 (0.5)	63.4 (0.5)	37.4 (0.3)	26.0 (0.8)	0.69
*W. globosa* 5537	32.6 (0.6)	4.11 (2.7)	63.3 (0.3)	36.8 (0.3)	26.5 (0.4)	0.72
*W. globosa* 9498	42.0 (0.9)	3.49 (0)	54.5 (0.7)	28.1 (0.7)	26.4 (0.8)	0.94
*W. microscopica* 2005	33.3 (3.6)	1.79 (1.1)	65.0 (1.8)	37.1 (1.9)	27.9 (2.5)	0.75
*W. neglecta* 9149	33.0 (1.5)	3.65 (0.8)	63.3 (0.9)	35.4 (1.1)	27.9 (0.7)	0.79
Average	33.9	3.47	62.6	37.2	25.4	0.70
± SD	3.1	1.3	3.2	4.1	2.5	0.14

*Data were given as average of the sums of specific fatty acids (cf. Tables 3, 4 for the single fatty acid content). As matrix-specific accuracy (RSD) does not exist, relative standard deviations (%) of the single sum of fatty acids were given in brackets. For averages ± SD cf. Table 2. SFA, Saturated fatty acids; MUFA, Monounsaturated fatty acids; PUFA, Polyunsaturated fatty acids*.

**Figure 3:**
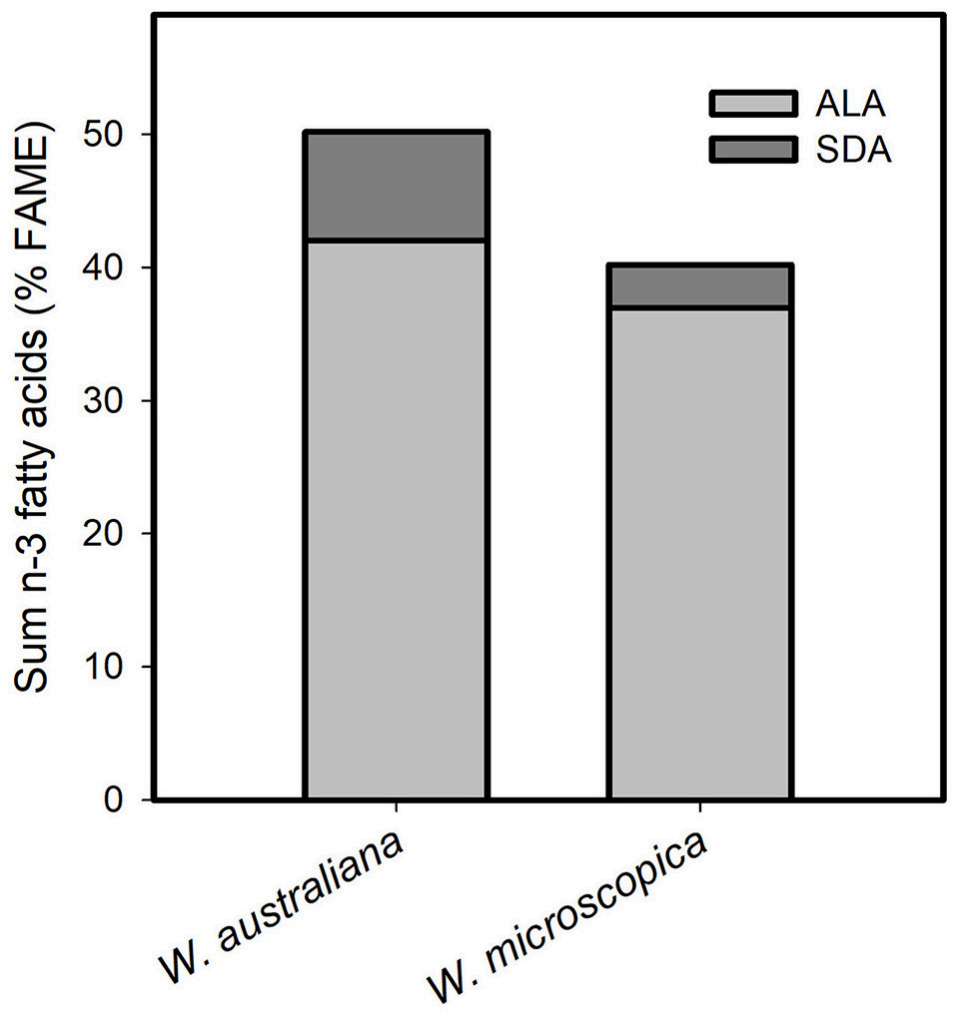
Content of the fatty acids α-linolenic acid (ALA) and stearidonic acid (SDA) in the two species *W. australiana* 7540 and *W. microscopica* 2005. Data were given as sum of the two *n*-3 fatty acids in relation to fatty acid methyl ester (FAME, %).

Most importantly (Table 5), the FA profile was dominated by polyunsaturated fatty acids (PUFA) with ~54% in *W. globosa* 9498 and ~68% in *W. arrhiza* 9528 (average across all *Wolffia* species: ~63%). The major PUFA was α-linolenic acid (ALA, C18:3-c9,12,15; n-3 fatty acid), followed by linoleic acid (LA, C18:2-c9,12; n-6 fatty acid). γ-Linolenic acid (GLA; C18:3-c6,9,12; n-6 fatty acid) and C20:2-c11,14 (11,14-eicosadienoic acid) showed very low abundance (Table 4). The amount of ALA was always higher than the sum of LA and GLA. Therefore, the important n-6/n-3 PUFA ratio was consistently < 1, ranging from 0.48 in *W. australiana* 7540 to 0.94 in *W. elongata* 9188 and *W. globosa* 9498 (Table 5). This was demonstrated by partially large RSDs of the averages (Tables 3–5). Stearidonic acid (SDA; n-3 PUFA) was solely detected in *W. microscopica* 2005 and in *W. australiana* 7540 (Figure 3) but was below the detection limit in all other clones.

### Minerals

The total ash content amounted to ~18% of FDW. The macro elements calcium, potassium, sodium, magnesium, iron and phosphorous (Table 6) as well as the microelements and non-essential heavy metals (Tables 7, 8) mercury, arsenic, selenium, copper, manganese, zinc, iodine, and lead were measured in all 16 duckweed samples. As the mineral content can be easily adjusted to the specific requirements of human nutrition by changing the composition of the nutrient medium (Appenroth et al., 2017), we focused here on the enrichment of minerals in different plants under identical growth medium conditions. Magnesium content ranged from 1.91 to 4.55 g/kg FDW (mean 2.85 ± 0.71 g/kg FDW), iron from 0.11 to 0.4 g/kg FDW (mean 0.23 ± 0.09 g/kg FDW), and the trace element manganese from 78.4 to 431 mg/kg FDW (mean 230 ± 98 mg/kg FDW). Interestingly, iodine ranged from 0.20 to 0.92 mg/kg FDW (mean 0.39 ± 0.19 mg/kg FDW), and cadmium content from 0.009 to 0.59 mg/kg FDW (mean 0.076 ± 0.145 mg/kg FDW), although both elements were not applied by purpose and must have been introduced as impurities of chemicals or water. It should be stated here that *pro analysi* chemicals and purified and desalted water (< 0.1 μS/cm) was used for growth medium preparation. Consequently, the uptake of minerals and trace elements also depends on the plant species studied.

**Table 6:** Macro elements and ash content [g/kg FDW] in species of *Wolffia*.

**Clone**	**Ca**	***K***	**Mg**	**Na**	***P***	**Ash content**
*W. angusta* 8878	14.0	46.7	2.2	0.25	12.1	127
*W. arrhiza* 8618	25.7	88.1	2.39	0.18	18.8	229
*W. arrhiza* 8853	23.1	62.9	3.06	0.38	16.5	174
*W. arrhiza* 9528	19.8	93.7	2.66	0.34	16.7	224
*W. australiana* 7540	14.1	87.6	2.8	0.37	12.0	209
*W. borealis* 9123	18.1	66.3	2.59	0.17	13.4	164
*W. brasiliensis* 7925	13.9	84.1	2.32	0.12	14.5	190
*W. columbiana* 7155	26.0	82.4	2.74	0.66	20.9	216
*W. cylindracea* 9056	22.1	93.8	3.46	0.26	17.2	222
*W. elongata* 9188	32.5	61.5	4.55	0.22	21.3	198
*W. globosa* 5514	25.2	52.7	3.17	0.12	16.7	160
*W. globosa* 5515	24.1	54.6	2.99	0.11	17.1	159
*W. globosa* 5537	21.3	52.3	2.77	0.11	16.9	147
*W. globosa* 9498	13.4	42.1	1.96	0.13	13.5	105
*W. microscopica* 2005	20.6	76.8	4.07	0.38	18.9	206
*W. neglecta* 9149	12.2	39.7	1.91	0.13	12.5	166
RSD (%)	3.2	3.6	3.0	4.1	2.0	0.44
Average	20.4	67.8	2.85	0.25	16.2	181
± SD	5.8	18.8	0.71	0.15	3.0	37

*For further explanations see Table 2*.

**Table 7:** Content of microelements in species of *Wolffia* [mg/kg FDW].

**Clone**	**Fe**	**Mn**	**Cu**	**Zn**	**I**	**Se**
*W. angusta* 8878	0.26	215	3.69	70.6	0.45	< 0.030
*W. arrhiza* 8618	0.16	78.4	3.31	50.0	0.31	< 0.030
*W. arrhiza* 8853	0.29	147	2.70	22.5	0.20	< 0.030
*W. arrhiza* 9528	0.20	144	2.89	40.7	0.25	< 0.030
*W. australiana* 7540	0.16	275	1.49	26.2	0.28	< 0.030
*W. borealis* 9123	0.19	249	2.70	33.4	0.44	< 0.030
*W. brasiliensis* 7925	0.13	330	2.96	31.4	0.20	< 0.030
*W. columbiana* 7155	0.13	80.4	3.43	69.6	0.92	< 0.030
*W. cylindracea* 9056	0.28	431	2.88	55.8	0.25	< 0.030
*W. elongata* 9188	0.31	336	2.34	65.8	0.51	< 0.030
*W. globosa* 5514	0.26	296	2.99	38.0	0.36	< 0.030
*W. globosa* 5515	0.12	302	4.43	41.2	0.20	< 0.030
*W. globosa* 5537	0.11	255	2.32	47.3	0.50	< 0.030
*W. globosa* 9498	0.37	203	2.50	84.2	0.50	< 0.030
*W. microscopica* 2005	0.33	136	3.83	79.4	0.56	< 0.030
*W. neglecta* 9149	0.40	200	2.39	92.4	0.27	< 0.030
RSD (%)	4.0	3.3	3.8	3.9	5.2	12.2
Average	0.23	230	2.93	53.0	0.39	< 0.030
± SD	0.09	98	0.70	21.7	0.19	–

*For further explanations see Table 2*.

**Table 8:** Heavy metals including As in species of *Wolffia*.

**Clone**	**Cd**	**Pb**	**Hg**	**As**
*W. angusta* 8878	81	1020	13	31
*W. arrhiza* 8618	13	100	17	85
*W. arrhiza* 8853	8.7	57	13	26
*W. arrhiza* 9528	11	52	13	60
*W. australiana* 7540	9	13	21	69
*W. borealis* 9123	51	120	15	64
*W. brasiliensis* 7925	14	23	22	39
*W. columbiana* 7155	590	410	18	69
*W. cylindracea* 9056	18	28	13	76
*W. elongata* 9188	63	89	27	52
*W. globosa* 5514	18	79	14	74
*W. globosa* 5515	15	71	17	35
*W. globosa* 5537	120	93	21	42
*W. globosa* 9498	15	610	17	42
*W. microscopica* 2005	180	460	24	42
*W. neglecta* 9149	17	680	18	45
RSD (%)	5.6	7.5	8.0	6.3
Average	76	244	18	53
± SD	145	301	4	18

*For definition of the lead-group of heavy metals, cf. Appenroth (2010). Data are given in μg/kg FDW. For further explanations see Table 2*.

### Carotenoids and tocopherols

In all 16 duckweed samples the contents of (*all-E*)-β-carotene, (*9Z*)-β-carotene, (*13Z*)-β-carotene, (*all-E*)-lutein, (*all-E*)-zeaxanthin, and α-tocopherol were analyzed (Table 9). The dominating carotenoid in all cases was clearly lutein (ca. 40–80 mg/100 g FDW), followed by (*all-E*)-β-carotene (ca. 10–30 mg/100 g FDW). The other carotenoids were present in much lower concentrations. The α-tocopherol content was between 0.5 and 13 mg/100 g FDW. A large variety of the contents of carotenoids and α-tocopherol in the different *Wolffia* species was observed as demonstrated by high standard deviations of the averages (Table 9). Impressive were the different contents of carotenoids and α-tocopherol in the different *Wolffia* species. For example, α-tocopherol content in *W. arrhiza* 8618 was ca. 13 mg/100 g FDW, while most other plants had contents of α-tocopherol lower than 5 mg/100 g FDW.

**Table 9:** Carotenoid and α-tocopherol contents in species of *Wolffia* [mg/100 g FDW] n.d., not detectable (< 0.2 mg/100 g FDW).

**Clone**	**(*all-E*)-β-Carotene**	**(*9Z*)-β-Carotene**	**(*13Z*)-β-Carotene**	**(all-E)-Lutein**	**(*all-E*)-Zeaxanthin**	**α-Tocopherol**
*W. angusta* 8878	16.7	3.5	0.85	59.5	2.1	2.9
*W. arrhiza* 8618	23.8	5.7	6.50	70.2	2.1	12.8
*W. arrhiza* 8853	27.3	5.8	1.43	62.0	2.9	3.3
*W. arrhiza* 9528	17.2	3.8	0.84	51.8	2.1	7.3
*W. australiana* 7540	29.7	6.5	1.76	79.4	2.1	3.2
*W. borealis* 9123	17.8	3.9	0.98	53.3	1.54	0.82
*W. brasiliensis* 7921	19.9	4.4	1.00	47.5	2.1	2.3
*W. columbiana* 7155	24.8	5.4	1.28	75.2	3.5	2.7
*W. cylindracea* 9056	12.3	2.7	n.d.	51.2	1.91	5.6
*W. elongata* 9188	33.0	6.9	1.98	78.5	1.76	4.6
*W. globosa* 5514	19.1	4.1	1.00	58.1	1.85	4.1
*W. globosa* 5515	11.4	2.4	0.67	41.3	1.82	3.2
*W. globosa* 5537	17.6	3.7	1.03	46.4	1.89	7.9
*W. globosa* 9498	11.0	2.4	n.d.	43.3	1.59	3.5
*W. microscopica* 2005	20.6	4.5	1.10	66.7	1.64	0.51
*W. neglecta* 9149	16.1	3.4	1.01	59.3	1.41	3.5
RSD (%)	10.0	10.0	10.0	10.0	10.0	20.0
Average	19.9	4.3	1.5	59.0	2.0	4.3
± SD	6.4	1.4	1.5	12.2	0.5	3.0

*The limit of detection (LOD) was calculated as three times the noise signal of a blank. For further explanations see Table 2*.

### Phytosterols, phytol, and dihydrophytol

Total sterol content was between 2.4 and 5.3% of the total fat content in the *Wolffia* samples (Table 10). The main component of sterols was clearly β-sitosterol ranging from 57 to 84% of the total sterols. The contents of campesterol (mean: 8.5% of total sterols) and stigmasterol (mean: 7.7% of total sterols) were much lower but the differences between the different plant samples were very large as indicated by large standard deviations of the averages. Stigmasterol could not be detected in *W. arrhiza* 8618 but had a share of 20% of the total sterols in *W. australiana* 7540. It is worth mentioning that also the other phytosterols, although in most cases present only in low amounts, showed very high variations between the different *Wolffia* species. Variation among the duckweed samples concerning the phytosterol content could not only be observed between the different species but also between the different clones of *W. arrhiza* (e.g., stigmasterol) and *W. globosa* (e.g., campesterol), although all plants were cultivated under identical conditions. During analysis of the phytosterol fraction we also noted two abundant peaks in the early part of the GC/MS chromatogram which were identified as 3,7*R*,11*R*,15-tetramethylhexadec-2E-enol (phytol) and the related dihydrophytol (Schröder et al., 2014). The isoprenoid alcohol phytol is usually bound to chlorophyll, which was also the source in the present samples. Concentrations of phytol (1.8–5.2% of the total fat content in the *Wolffia* samples) were in the range of the phytosterols, while the content of dihydrophytol was around 1% or lower (Table 10). Phytol has been detected in various plant oils but never at concentrations higher than 50 mg/100 g oil (Schröder et al., 2014).

**Table 10:** Sterols and phytols in *Wolffia* species.

**Clone**	**Total sterols**	**Phytol**	**Dihydro-phytol**	**Campesterol**	**Stigmasterol**	**β-Sitosterol**	**Sitostanol**	**Δ5-Avena-sterol**	**Δ7-Sitosterol**	**24-Methylene cycloartanol**
*W. angusta* 8878	2.8	3.4	0.2	11	12	75	n.d.	2	n.d.	n.d.
*W. arrhiza* 8618	5.2	1.8	n.d.	9	n.d.	84	7	n.d.	n.d.	n.d.
*W. arrhiza* 8853	4.3	3.9	0.7	8	5	73	14^a^	n.q.	n.d.	n.d.
*W. arrhiza* 9528	4.6	4.8	0.5	11	2	77	10^a^	n.q.	n.d.	n.d.
*W. australiana* 7540^*^	3.8	4.9	0.5	7	20	61	n.d.	6	n.d.	2
*W. borealis* 9123	3.4	4.5	1.3	8	4	81	n.d.	3	n.d.	4
*W. brasiliensis* 7925	3.7	5.2	n.d.	7	17	73	n.d.	3	n.d.	n.d.
*W. columbiana* 7155^*^	4.4	4.7	n.d.	5	1	73	n.d.	n.q.^b^	17	n.d.
*W. cylindracea* 9056^*^	4.6	3.6	0.4	8	1	77	11^a^	n.q.	n.d.	2
*W. elongata* 9188^*^	5.3	3.8	0.7	13	17	57	n.d.	n.q.^b^	n.d.	7
*W. globosa* 5514	3.8	4.8	0.5	7	4	79	n.d.	3	2	5
*W. globosa* 5515	3.2	2.5	0.6	6	5	78	n.d.	2	3	6
*W. globosa* 5537	3.8	3.3	1.1	5	3	82	n.d.	3	2	5
*W. globosa* 9498	2.4	2.3	1.1	12	5	77	n.d.	6	n.d.	n.d.
*W. microscopica* 2005^*^	3.6	3.9	0.5	12	15	60	n.d.	9	n.d.	3
*W. neglecta* 9149^*^	3.9	3.0	0.5	7	4	80	n.d.	4	n.d.	n.d.
RSD (%)	7	6	4	4	4	1	3	5	3	3
Average	4.1	3.9	0.9	8	7	70	9	4	5	4
± SD	1.1	1.1	0.9	3	6	19	4	2	6	2

*Total sterols, phytol and dihydrophytol are given in g/100 g fat. LOD = 0.007 g/100 g fat, LOQ = 0.02 g/100 g fat. Sterol components were given as % of total sterols. n.d., not detectable; n.q., not quantifiable. Limit of detection (LOD) and Limit of quantification (LOQ) were calculated as three and ten times the noise signal of a blank. For further explanations see Table 2*.

a *Coelution with Δ5-avenasterol*,

b Coelution with β-amyrin (~4–5 mg/g fat)

* further minor sterols contributing up to 6% to the total sterol content which are not listed here were 24- methylene cholesterol, cycloartenol, Δ7-stigmastenol and Δ5,24(25)-stigmastadienol

Method precision < 10%, calculated for each substance as relative standard deviation (RSD) within the actual measurement concentration (n_sample_ = 2; 2 ≤ n_batch_ ≤ 16) $RSD_{batch}\left[\%\right]=\;\bar{RSD_{sample}}=\;\frac{1}{n_{batch}}\overset{n_{batch}}{\underset{i=1}{\sum }}\frac{SD_{sample}}{\bar{x}}\;\cdot 100$

## Discussion

In our recently published study (Appenroth et al., 2017), we investigated the protein, fat and starch contents, amino acid and fatty acid distribution of six duckweed species encompassing all five genera (Sree et al., 2016) in order to get an overview of the nutritional properties of duckweeds. We selected *W. microscopica* 2005 to investigate also the content of minerals, carotenoids and α-tocopherol as well as phytosterols and fiber. Knowledge of the composition of these plants is essential for potential users for human nutrition and to fulfill the judicial requirements of the novel food regulation during applications in the future. In order to deepen our understanding of the nutritional value of these tiny plant species, we selected 16 clones, all belonging to the genus *Wolffia*, comprising all the eleven species known till-date and investigated the above-mentioned components in the duckweed samples. This makes it possible to search for those *Wolffia* species or even clones of the same species that may represent valuable food sources for human nutrition.

In line with our previous investigation (Appenroth et al., 2017) reporting about the total protein content of the six species belonging to the five genera, the total protein content of the *Wolffia* species analyzed in the present study was in the similar range. *W. microscopica* 2005 was investigated in both projects and it contained slightly less protein and slightly more starch in the present study than in the recently published (Appenroth et al., 2017). This might be due to the longer cultivation period and the modification of growth medium. The fat content was low in general, but in contrast to FDW, starch, and fiber, the fat content varied between the different species. Even clones of the same species, such as *W. arrhiza* and *W. globosa*, showed significant differences in the total fat content. However, more important than the differences in the quantity of the different biochemical components, is the nutritional quality of these components. In our previous publication (Appenroth et al., 2017), we compared already the quality and quantity of nutritional components in several duckweeds with other plant species (cf. also Edelman and Colt, 2016) and demonstrated the advantages of duckweed especially concerning protein and fat quality.

### Protein content and amino acid composition

In the set of 16 *Wolffia* samples that were investigated, *W. microscopica* played a special role by having a high protein quality with respect to human nutrition (cf. Appenroth et al., 2017). In comparison to the suggested reference values for essential amino acid requirements of preschool-age children [WHO (World Health Organization), 1985, 2007], all species and clones of *Wolffia* studied here showed excellent protein qualities. All ratios between the measured AA content and the reference (Figure 2 and Table S2) were above 1 or close to 1 and *W. microscopica* is a top-scoring species. Interestingly, there are also differences between the different clones of *W. globosa* and *W. arrhiza*. Thus, we can also recommend the selection of more suitable clones for human nutrition.

Amado et al. (1980) published the most comprehensive screening for total protein content and amino acid composition of 26 species in a preliminary report. Beside a large number of clones of all other genera, the authors studied 24 clones of seven *Wolffia* species out of the nine species known at that time (Landolt, 1980; Sree et al., 2016). The authors reported analytical difficulties for the determination of sulfur-containing AA, which might explain why they called their study preliminary. Taken together, the findings of Amado et al. (1980) and Appenroth et al. (2017), and the study presented here cover the genus *Spirodela* with 11 clones, *Landoltia* with five clones, *Lemna* with 39 clones, *Wolffiella* with 20 clones and *Wolffia* with 40 clones. Together, it can be concluded that a high protein content and the quality of the amino acid spectrum of duckweed makes many of these plants suitable for human nutrition.

### Fat content and fatty acid distribution

We have recently shown that the quality of the fatty acid profile of *Wolffia* covers nicely the requirements of human nutrition, although the fat content is generally rather low (Appenroth et al., 2017). In the present study, we found that the contribution of PUFA was 60% or higher for all analyzed *Wolffia* species, except for *W. globosa* 9498 (~54.5% PUFA). Tang et al. (2015) investigated the fatty acid contents of the four species, *Spirodela polyrhiza, Landoltia punctata, Lemna aequinoctialis*, and *W. globosa* isolated from the lake Chao, China. Yan et al. (2013) reported the most comprehensive survey of fatty acids in duckweeds by investigating 30 species (one clone per species) including eight species of *Wolffia*. Unfortunately, the plants were cultivated mixotrophically (i.e., in the presence of sugar), which increases evidently the total fat content but makes it practically useless in a biotechnological context (Appenroth et al., 2017).

The main PUFA in the *Wolffia* species was the n-3 PUFA ALA with contents ranging from 28 to 43% FAME (Table 4). The content of the n-3 PUFA SDA is also worth mentioning (Figure 3). This fatty acid is of high importance for human nutrition; it is already Δ-6 desaturated, has four double bonds and can be improved, i.e., metabolized, to the long-chain n-3 PUFA eicosapentaenoic acid (C20:5-c5,8,11,14,17) and docosapentaenoic acid (C22:5-c,4,7,10,13,16) in humans (Kuhnt et al., 2014, 2016; Dittrich et al., 2015). Within the genus *Wolffia* we detected SDA only in *W. microscopica* 2005 and *W. australiana* 7540. This makes these two species interesting for human nutrition as well as fundamental for nutrition research. In humans, the long-chain n-3 PUFA are also metabolized from ALA, which is the major fatty acid in the lipid fraction of the studied *Wolffia* species, whereby the conversion from SDA is more effective (Dittrich et al., 2015). The long-chain n-3 PUFA acts as anti-inflammatory and can therefore support the therapy of chronic inflammatory diseases such as rheumatoid arthritis (Dawczynski et al., 2017).

The major n-6 PUFA detected in the *Wolffia* species were LA and GLA, both fatty acids together represent nearly 30% FAME (Table 4). Beside the PUFA content, the n-6/n-3 ratio is important for human nutrition, because there is an imbalance marked by high intake of n-6 PUFA from plant oils such sunflower and soya oil, grains, sausage, and meat. On the other hand, the intake of n-3 PUFA do not reach the recommendations. This problem is of particular importance for the increasing number of vegetarians and vegans, which ban fish and meat.

Due to the enormous ALA content, the n-6/n-3 ratio was below 1.0 in all *Wolffia* species studied. According to the recommendations of the Food and Agriculture Organization of the United Nations (FAO, 2010), the n-6/n-3 ratio should not be >5 in human nutrition. Simoupolos (2006) reported that in Western diets the n-6/n-3 ratio is usually at least 15:1 to 17:1. A high n-6/n-3 ratio promotes the pathogenesis of many diseases, including cardiovascular diseases, cancer, and osteoporosis as well as inflammatory and autoimmune diseases. Thus, the generally high proportions of n-3 fatty acids of duckweed (Appenroth et al., 2017) and detected in all *Wolffia* species analyzed in this study could lower this ratio and therefore may contribute to a healthier human diet. *W. globosa*, which has a lower n-6/n-3 ratio than most other *Wolffia* species, is already used for human nutrition in several Asian countries.

### Starch and fiber content

The starch content of the *Wolffia* species investigated in this study was 10 to 15% and is higher than in our previous study (Appenroth et al., 2017). This is, at least in part, caused by the slightly different cultivation conditions (i.e., especially longer cultivation time), as even the clone *W. microscopica* 2005 had higher starch content in the present study than measured before. However, the four clones of *W. globosa* showed different starch content although the cultivation conditions were identical. Therefore, the genetic constitutions must also influence starch production and accumulation. In summary, the starch content was low in all plant samples investigated, including the clones of *W. globosa*, which is already used for human nutrition. This low content of starch fits the requirements in Western countries to reduce high carbohydrate intake. In several developing countries, the low starch content has no disadvantages as human food, as starch intake is often high in these countries because of the staple food rice and maize [EFSA (European Food Safety Authority), 2017].

The fiber content in the investigated samples was similar and amounted to 26.2 ± 1.2% of FDW (Figure 1). This high content of dietary fiber is beneficial with respect to improving the Western diet by low-energy food components. Moreover, a high intake of dietary fiber is associated with a control of cardiovascular risk factors, such as total cholesterol and LDL cholesterol, and will therefore contribute to prevention and therapy of cardiovascular diseases, which are the major cause of death in the European countries (Kelly et al., 2017).

### Mineral composition

The mineral composition of all *Wolffia* samples investigated can be characterized as relatively rich in potassium and iron, and poor in sodium, making the duckweeds useful for healthy human nutrition. Duckweed samples contained the trace elements manganese, zinc and copper, although copper was not supplied through the nutrient medium. It might have accumulated from the impurities of supplemented macronutrients. The contents of minerals can be varied within a wide range by the application of suitable concentrations of the mineral content in the nutrient medium. This effect has not yet been investigated systematically in the context of optimizing intake of mineral nutrients for humans, but it can be expected that plants with high contents of selenium, zinc, or iodine can be easily produced with optimized nutrient media. Interestingly, different clones of the same species had different mineral contents although cultivated under identical conditions. For example, the iron content ranged between 0.11 and 0.37 g/kg FDW in the different clones of *W. arrhiza* and between 0.10 and 0.29 g/kg FDW in *W. globosa*. This means that also a genetic influence exists, resulting in different contents of trace elements and minerals under identical cultivation conditions.

### Carotenoids and vitamin E

The main carotenoids (*all*-*E*)-lutein and (*all*-*E*)-β-carotene, showed remarkably high contents and variations among the different *Wolffia* species. Together with the high values of zeaxanthin, this finding is important for the prevention of age-related macular degeneration, especially in comparison with other vegetarian food sources (Chew et al., 2013; Westphal and Böhm, 2015). *Wolffia australiana* 7540 and *W. elongata* 9188 showed the highest contents of both lutein and β-carotene. The content of α-tocopherol varied in the different species. The highest value was measured in *W. arrhiza* 8618, whereas the two other clones of this species had either medium (*W. arrhiza* 9528) or even low (*W. arrhiza* 8853) contents of α-tocopherol, again indicating high intraspecific genetic variation. Further, the role of light intensity or photoperiod remains to be investigated in this context in *Wolffia* species.

### Phytosterols

Phytosterols have several effects on human health (Kritchevsky and Chen, 2005; Jahreis et al., 2013). Our results show that *Wolffia* species are a rich source of phytosterols, such as β-sitosterol, campesterol, and stigmasterol, which contribute to the valuable nutrient profile of duckweeds. Moreover, it is evident that there are large differences between the different *Wolffia* species. This holds true especially for the minor components 24-methylenecholesterol, Δ5-avenasterol, Δ5,24(25)-stigmadienol, Δ7-sitosterol, Δ7-stigmasterol, cycloartenol and 24-methylenecycloartanol. *W. arrhiza* 8618 had the highest content of β-sitosterol, *W. australiana* 7540 had the highest content of stigmasterol, *W. elongata* 9188 had the highest content of campesterol and *W. arrhiza* 8853 had the highest levels of sitostanol and Δ5-avenasterol. In comparison with other *Wolffia* species, there seems to be no specific advantage of *W. globosa*, which is widely used for human nutrition in some Asian countries. The three investigated clones of *W. arrhiza* and the four clones of *W. globosa* illustrate that the content of phytosterols also depends on the origin of the clones and not only on the species.

### Yield of nutritional components

For the intended practical application as human food, growth rates of the *Wolffia* plants as given in Table 1 are important (see also Sree et al., 2015). As the yields of dry weight indicate, the differences were large and ranged from 5.4 g per g per week to almost 130 g per g per week. The high content of dietary fiber is beneficial in improving the Western diet by adding low-energy food components. Consequently, the plants with the highest growth rate have also the highest yield in protein, fat and starch (Supplementary Table S3). The dominating plant is *W. microscopica* 2005, as already shown in our previous publication (Appenroth et al., 2017). This species represents the fastest growing angiosperm of all (Sree et al., 2015; Sree et al., unpublished). There were also large differences in the yield between clones of the same species, i.e., *W. arrhiza* and *W. globosa* (Sree et al., 2015). In summary, our data show that it is worth to extend the investigations on *Wolffia* species including different clones to evaluate their potential for practical applications as human food.

## Author contributions

K-JA, KSS, and GJ drafted the project. GJ coordinated analyses and evaluation of the data. K-JA and KSS produced the plant material. K-JA and GJ wrote the first draft of the manuscript. KSS analyzed the starch content. MB identified the plant species and measured growth rates. JE, SL, CD, CS, and GL analyzed and evaluated the fatty acid content. VB analyzed tocopherol and carotenoids. KS and WV analyzed phytosterols and phytols. KT-B and ML analyzed amino acids. RK and ML minerals. All authors read and revised the manuscript and agreed with the text.

### Conflict of interest statement

The authors declare that the research was conducted in the absence of any commercial or financial relationships that could be construed as a potential conflict of interest.

## Abbreviations

AA: amino acids
Ala: alanine
ALA: α-linolenic acid
Arg: arginine
Asp: aspartic acid
Cys: cysteine
DW: dry weight
EAA: essential amino acids
FA: fatty acids
FAO: Food and Agriculture Organization of the United Nations
FAME: fatty acid methyl esters
FDW: freeze-dry weight
GLA: gamma-linolenic acid
Glu: glutamic acid
Gly: glycine
Ile: isoleucine
LA: linoleic acid
LCFA: long-chain fatty acids
Leu: leucine
LDL: Low density lipoprotein
Lys: lysine
Met: methionine
MUFA: monounsaturated fatty acids
Phe: phenylalanine
PUFA: polyunsaturated fatty acids
SCFA: short-chain fatty acids
SD: standard deviation
SDA, stearidonic acid (C18:4c6, 9, 12: 15)
SFA: saturated fatty acids
Thr: threonine
Trp: tryptophan
Val: valine
WHO: World Health Organization.
