# Supplementary material for: Nutritional Value of the Duckweed Species of the Genus Wolffia (Lemnaceae) as Human Food
Source: Front Chem. 2018 Oct 29;6:483. doi: 10.3389/fchem.2018.00483 (PMC6215809; doi:10.3389/fchem.2018.00483)
Supplement: Supplementary file 1 [file Table_1.DOCX]

Supplementary Material:

**Supplementary Table S1A**: Identification of *Wolffia* species by sequencing plastidic fragments: *rpl*16, *rps*16, *atp*FH or *psb*KI.

| **Clone** | ***rpl*16** | ***rps*16** | ***atp*FH** | ***psb*KI** |
| --- | --- | --- | --- | --- |
| *Wolffia angusta* 8878 | HE819921 | HE819976 |  |  |
| *Wolffia arrhiza* 8618 |  |  | MG812317 |  |
| *Wolffia arrhiza* 8853 | HE819928 | HE819983 |  |  |
| *Wolffia arrhiza* 9528 |  |  | MG812318 |  |
| *Wolffia australiana* 7540 | HE819931 | HE819986 |  |  |
| *Wolffia borealis* 9123 |  | HE819989 | GU454270 | GU454367 |
| *Wolffia brasiliensis* 7925 | HE819937 | HE819993 | MG812319 |  |
| *Wolffia columbiana* 7155 | HE819942 | HE819998 | MG812320 |  |
| *Wolffia cylindracea* 9056 | HE819949 |  | MG812321 | MG812326 |
| *Wolffia elongata* 9188 | HE819951 | HE820006 | GU454280 | GU454377 |
| *Wolffia globosa* 5514 |  |  | MG812322 | MG812327 |
| *Wolffia globosa* 5515 |  |  | MG812323 | MG812328 |
| *Wolffia globosa* 5537 |  |  | MG812324 | MG812329 |
| *Wolffia globosa* 9498 | HE819965 | HE820020 | MG812325 | MG812330 |
| *Wolffia microscopica* 2005 |  |  | KJ921752 | KJ921766 |
| *Wolffia neglecta* 9149 |  |  | GU454289 | GU454386 |

**Supplementary Table S1 B:** Identification of *Wolffia* species by best BLAST hit

| **Clone** | **Marker** | **Best BLAST hit** |
| --- | --- | --- |
| *Wolffia arrhiza* 8618 | *atp*HF | *Wolffia arrhiza* strain 8872 *atp*H-like gene, partial sequence; and ATP synthase subunit I (*atp*F) gene, partial cds |
| *Wolffia arrhiza* 9528 | *atp*HF | *Wolffia arrhiza* strain 8872 *atp*H-like gene, partial sequence; and ATP synthase subunit I (*atp*F) gene, partial cds |
| *Wolffia brasiliensis* 7925 | *atp*HF | *Wolffia brasiliensis* strain 7306 atpH and ATP synthase subunit I (*atp*F) genes, partial cds; chloroplast |
| *Wolffia columbiana* 7155 | *atp*HF | *Wolffia columbiana* strain 8856 *atp*H-like gene, partial sequence; and ATP synthase subunit I (*atp*F) gene, cds |
| *Wolffia cylindracea* 9056 | *atp*HF | *Wolffia cylindracea* strain 9080 *atp*H-like gene, partial sequence; and ATP synthase subunit I (*atp*F) gene, cds |
|  | *psb*KI | *Wolffia cylindracea* strain 9080 *psb*KI intergenic spacer, partial sequence; chloroplast |
| *Wolffia globosa* 5514 | *atp*HF | *Wolffia globosa* strain DW2101-4 ATPase subunit I (*atp*F) gene, partial cds; *atp*FH intergenic spacer, complete sequence; and ATPase subunit III (*atp*H) gene, partial cds; chloroplast |
|  | *psb*KI | *Wolffia globosa* strain 8152 *Psb*K (*psb*K) gene, partial cds; and *psb*KI intergenic spacer, partial sequence |
| *Wolffia globosa* 5515 | *atp*HF | *Wolffia globosa* strain DW2101-4 ATPase subunit I (*atp*F) gene, partial cds; *atp*FH intergenic spacer, complete sequence; and ATPase subunit III (*atp*H) gene, partial cds; chloroplast |
|  | *psb*KI | *Wolffia globosa* strain 8152 PsbK (*psb*K) gene, partial cds; and *psb*KI intergenic spacer |
| *Wolffia globosa* 5537 | *atp*HF | *Wolffia globosa* strain DW2801-3 ATPase subunit I (*atp*F) gene, partial cds; *atp*FH intergenic spacer, complete sequence; and ATPase subunit III (*atp*H) gene, partial cds; chloroplast |
|  | *psb*KI | *Wolffia globosa* strain 8152 PsbK (*psb*K) gene, partial cds; and *psb*KI intergenic spacer, partial sequence |
| *Wolffia globosa* 9498 | *atp*HF | *Wolffia globosa* strain DW2101-4 ATPase subunit I (*atp*F) gene, partial cds; *atp*FH intergenic spacer, complete sequence; and ATPase subunit III (*atp*H) gene, partial cds; chloroplast |
|  | *psb*KI | *Wolffia borealis* strain 9147 *psb*KI intergenic spacer, partial sequence; plastid |
|  | *psb*KI | *Wolffia globosa* strain 8789 PsbK (*psb*K) gene, partial cds; and *psb*KI intergenic spacer, partial sequence |

**Supplementary Table S2** Essential amino acid (EAA) content in duckweed clones and requirements for essential amino acids in preschool-age children (FAO/WHO/UNU 1985). EAA content given as g/100 g protein, ratio of measured EAA content with that of reference is provided in parenthesis. Ratios < 1 are given in bold. For the calculation of the means and relative standard deviations, see Table 2. C+M: cysteine + methionine; H+F: histidine + phenylalanine.

| **EAA**  **Clone** | **Ile** | **Leu** | **Lys** | **C+M** | **H+F** | **Thr** | **Val** |
| --- | --- | --- | --- | --- | --- | --- | --- |
| **Reference**  **WHO, 1985** | **2.8** | **6.6** | **5.8** | **2.5** | **6.3** | **3.4** | **3.5** |
| *W. angusta* 8878 | 3.9 (1.4) | 8.4 (1.3) | 6.0 (1.03) | 3.4 (1.4) | 6.7 (1.1) | 4.4 (1.3) | 5.0 (1.4) |
| *W. arrhiza* 8618 | 3.8 (1.4) | 8.3 (1.3) | 6.0 (1.03) | 2.9 (1.2) | 7.1 (1.2) | 4.0 (1.2) | 5.0 (1.4) |
| *W. arrhiza* 8853 | 3.3 (1.2) | 7.2 (1.1) | 5.4 (**0.93**) | 2.9 (1.2) | 6.1 (**0.97**) | 3.8 (1.1) | 4.4 (1.3) |
| *W. arrhiza* 9528 | 3.3 (1.2) | 7.1 (1.1) | 4.9 (**0.84**) | 2.8 (1.1) | 6.0 (**0.95**) | 3.6 (1.1) | 4.5 (1.3) |
| *W. australiana* 7540 | 3.6 (1.3) | 7.7 (1.2) | 5.6 (**0.97**) | 2.9 (1.2) | 6.6 (1.1) | 4.1 (1.2) | 4.7 (1.3) |
| *W. borealis* 9123 | 3.8 (1.4) | 8.0 (1.2) | 6.0 (1.03) | 3.1 (1.2) | 6.9 (1.1) | 4.2 (1.2) | 5.1 (1.5) |
| *W. brasiliensis* 7925 | 3.7 (1.3) | 8.1 (1.2) | 6.0 (1.03) | 3.1 (1.2) | 6.8 (1.1) | 4.0 (1.2) | 5.1 (1.5) |
| *W. columbiana* 7155 | 3.2 (1.1) | 7.2 (1.1) | 5.2 (**0.90**) | 3.0 (1.2) | 6.1 (**0.97**) | 3.4 (1.0) | 4.3 (1.2) |
| *W. cylindracea* 9056 | 3.6 (1.3) | 7.7 (1.2) | 5.9 (1.02) | 3.3 (1.3) | 6.4 (1.02) | 3.9 (1.1) | 4.8 (1.4) |
| *W. elongata* 9188 | 3.9 (1.4) | 8.6 (1.3) | 5.7 (**0.98**) | 3.1 (1.2) | 7.5 (1.2) | 4.6 (1.4) | 5.1 (1.5) |
| *W. globosa* 5514 | 3.7 (1.3) | 7.7 (1.2) | 5.4 (**0.93**) | 3.3 (1.3) | 6.7 (1.1) | 4.5 (1.3) | 4.8 (1.4) |
| *W. globosa* 5515 | 3.3 (1.2) | 6.9 (1.05) | 5.0 (**0.86**) | 3.0 (1.1) | 5.8 (**0.92**) | 3.9 (1.1) | 4.3 (1.2) |
| *W. globosa* 5537 | 3.6 (1.3) | 7.5 (1.1) | 5.4 (**0.93**) | 3.1 (1.2) | 6.3 (1.0) | 4.2 (1.2) | 4.6 (1.3) |
| *W. globosa* 9498 | 3.8 (1.4) | 7.7 (1.2) | 5.6 (**0.97**) | 3.1 (1.2) | 6.7 (1.1) | 4.5 (1.3) | 5.0 (1.4) |
| *W. microscopica* 2005 | 3.8 (1.4) | 8.1 (1.2) | 6.0 (1.03) | 3.3 (1.3) | 6.6 (1.05) | 4.5 (1.3) | 5.0 (1.4) |
| *W. neglecta* 9149 | 3.7 (1.3) | 7.9 (1.2) | 6.1 (1.1) | 3.3 (1.3) | 6.6 (1.1) | 4.3 (1.3) | 5.1 (1.5) |
| **Average** | **3.6 (1.3)** | **7.8 (1.2)** | **5.6 (0.97)** | **3.1 (1.2)** | **6.5 (1.03)** | **4.1 (1.2)** | **4.8 (1.4)** |
| **± SD (%)** | **6.3** | **6.5** | **6.9** | **5.8** | **6.6** | **8.4** | **6.2** |

WHO (World Health Organization). Energy and Protein Requirements. Report of a Joint FAO/WHO/UNU Expert Consultation. Technical Report Series 724. World Health Organization, Geneva, 1985, 206 pp.

**Supplementary Table S3.** Relative yield of dry weight, protein, fatty acids and starch produced by the given species of duckweed after cultivation with a starting material equivalent to 1 g of dry weight for 7 days. Results are given in g per g of initial dry weight. Ether extract is the total fat content. For average ± relative standard deviation see Table 2.

| **Species/ Clone** | **Freeze dry weight** | **Protein** | **Ether extract** | **Starch** |
| --- | --- | --- | --- | --- |
| *W. angusta* 8878 | 40.3 | 10.3 | 1.41 | 6.32 |
| *W. arrhiza* 8618 | 19.8 | 4.16 | 0.32 | 3.40 |
| *W. arrhiza* 8853 | 8.40 | 2.34 | 0.29 | 1.01 |
| *W. arrhiza* 9528 | 15.5 | 3.75 | 0.51 | 1.88 |
| *W. australiana* 7540 | 33.8 | 9.40 | 0.68 | 4.42 |
| *W. borealis* 9123 | 19.8 | 4.18 | 0.50 | 2.78 |
| *W. brasiliensis* 7925 | 34.0 | 8.47 | 1.77 | 3.77 |
| *W. columbiana* 7155 | 5.40 | 1.22 | 0.038 | 0.65 |
| *W. cylindracea* 9056 | 9.10 | 2.04 | 0.19 | 0.99 |
| *W. elongata* 9188 | 22.0 | 4.95 | 1.17 | 3.02 |
| *W. globosa* 5514 | 11.6 | 2.98 | 0.48 | 1.57 |
| *W. globosa* 5515 | 9.00 | 2.16 | 0.25 | 1.30 |
| *W. globosa* 5537 | 15.1 | 3.64 | 0.57 | 2.44 |
| *W. globosa* 9498 | 49.9 | 10.6 | 1.70 | 8.23 |
| *W. microscopica* 2005 | 126 | 36.7 | 2.77 | 13.23 |
| *W. neglecta* 9149 | 20.1 | 4.20 | 0.94 | 2.90 |
| **Average** | **27.5** | **6.94** | **0.85** | **3.62** |
| **CV (%)** | **106** | **122** | **87** | **90** |
